# Geospatial Insights into Healthcare Accessibility in Europe: A Scoping Review of GIS Applications

**DOI:** 10.3390/healthcare13222865

**Published:** 2025-11-11

**Authors:** Silviya Nikolova, Teodora Aleksandrova

**Affiliations:** Department of Social Medicine and Health Care Organisation, Medical University of Varna, 9000 Varna, Bulgaria; teodora.petrova@mu-varna.bg

**Keywords:** geographic information systems (GIS), healthcare, access, spatial analysis, public health, Europe

## Abstract

**Background:** Geographic Information Systems (GIS) have emerged as a critical tool in healthcare research, facilitating the assessment of healthcare accessibility through spatial analysis and data visualisation. This scoping review synthesises literature published between 2020 and 2024, a period marked by the COVID-19 pandemic and rapid methodological innovation, providing a timely overview of how GIS has been applied to evaluate healthcare access across European countries. **Methods:** The review underscores the role of GIS methodologies in identifying geographic disparities, optimising resource distribution, and informing policy decisions. **Results:** Key findings highlight significant urban-rural differences in healthcare access, shaped by factors such as transportation infrastructure, population density, and healthcare facility distribution. Additionally, GIS has proven valuable in examining the link between healthcare accessibility and utilisation, with better access generally correlating with higher service use. **Conclusions:** Despite its potential, challenges including data availability, methodological variability, and uneven adoption across regions limit its broader implementation. The review emphasises the need for integrating advanced technologies to foster more equitable healthcare access throughout Europe.

## 1. Introduction

Geographic Information Systems (GIS) have become an invaluable tool in healthcare research, offering innovative ways to assess healthcare accessibility. Defined as an information technology that stores, analyzes, and displays both spatial and non-spatial data including demographic characteristics, disease incidence, facility attributes, and socioeconomic indicators [1], GIS enables the visualisation of spatial patterns that are crucial for understanding and improving healthcare systems. By integrating geospatial data with health-related information, GIS helps map healthcare facilities, identify underserved areas, and assess the distribution of healthcare resources, providing evidence that supports more equitable allocation of services and guides policy decisions on improving healthcare access [2,3,4].

In the context of Europe, GIS has gained increasing attention as healthcare systems face the dual challenges of vulnerable populations and rising healthcare costs [5]. With the goal of ensuring more equitable and efficient service delivery, GIS-based analyses have generated insights into regional differences in accessibility and their implications for equity and system performance. For instance, studies have identified geographic disparities in service availability between urban and rural areas, thereby supporting data-driven policy decisions aimed at improving health equity and optimising resource allocation [6,7,8]. Rather than GIS data itself, it is the results of GIS analyses that allow tracking of disease prevalence and the identification of healthcare access inequalities, offering policymakers practical evidence for designing targeted interventions [9,10].

Specifically, GIS has been instrumental in evaluating healthcare accessibility by analysing geographic barriers to care, such as distance, transportation issues, and the availability of facilities. GIS allows for the optimisation of healthcare facility locations, ensuring that resources are allocated to areas in need, particularly in underserved or rural regions [11,12]. Furthermore, GIS is crucial for identifying healthcare inequalities, whether in terms of service availability or population demographics, and offers actionable insights into where inequities persist and how resources might be redistributed to address them [13,14,15].

Despite the potential benefits, the adoption of GIS in European healthcare systems remains relatively limited compared to other regions, such as the United States, where GIS has been more widely integrated [16,17,18,19,20]. This scoping review examines the extent and application of GIS-based analyses in evaluating healthcare accessibility across European countries between 2020 and 2024. By focusing on this recent period, the review captures developments influenced by the COVID-19 pandemic and reflects contemporary applications of GIS in healthcare research. It provides an updated synthesis of evidence, highlighting methodological approaches, regional disparities, and policy implications for healthcare accessibility in Europe. The review therefore aims to map how GIS has been applied in this period, identify emerging trends and persistent challenges, and assess the opportunities for enhancing healthcare access through GIS, with a view to informing both research and policy.

## 2. Materials and Methods

### 2.1. Study Design

This scoping review was conducted in accordance with the PRISMA-ScR guidelines [21]. This framework consists of five key stages: (1) defining the research question, (2) systematically identifying relevant studies, (3) applying eligibility criteria for study selection, (4) extracting and charting data using a structured approach, and (5) synthesising, summarising, and reporting findings. The review was designed to comprehensively map the existing literature and identify key themes related to GIS applications for healthcare accessibility in European countries. The review was restricted to studies published between January 2020 and December 2024 to ensure the inclusion of the most recent developments. This study is part of project 24025/2024 (Autumn session), funded by the Science Fund of the Medical University of Varna, Bulgaria.

### 2.2. Search Strategy

The search strategy was developed using terms related to GIS analysis and healthcare access in European countries. A combination of keywords was used, including “Geographical Information System,” “GIS,” “spatial analysis,” “healthcare accessibility,” “health service access,” “medical service access,” “health services,” “healthcare facilities,” “treatment centers,” “European countries,” “EU countries,” “EU member states,” “European Union member countries,” and “mapping,” “cartography,” “geomapping,” and “spatial mapping.” The names of all 53 countries in the European Region were included to expand the scope of the search. Boolean operators (AND; OR) were used to combine keywords, and the strategy was adjusted according to the database used. The search strategy was implemented in both PubMed and Google Scholar and was developed collaboratively by two researchers (T.A. and S.N.), who conducted the search process simultaneously, each applying the predefined search criteria independently. Both researchers independently performed searches using the same set of keywords and databases. Afterward, the results were compared, and any discrepancies were discussed to ensure consistency and accuracy in the selection of relevant articles. This approach allowed us to cross-check findings and refine the search strategy as needed, enhancing the robustness of the results and ensuring that no relevant articles were overlooked. The final search was conducted in December 2024, and for Google Scholar the first 200 results were screened for each query to ensure relevance.

### 2.3. Information Sources

The search was conducted in PubMed and Google Scholar using the defined keywords, focusing on articles published between 2020 and 2024. Scoping searches were performed to identify relevant articles already known to the review team. Additionally, citation searching of relevant articles was conducted to identify additional primary research articles for inclusion within this time period.

### 2.4. Research Question

Our research question was: “How has Geographic Information System (GIS) analysis been utilised to examine healthcare access and services in European countries?”. We used the Population, Concept, and Context (PCC) framework to determine the eligibility of our research question for this scoping review study, as shown in Table 1.

The eligibility criteria of this review included the following inclusion and exclusion criteria:

Inclusion Criteria:

- Studies utilising GIS or spatial analysis to examine healthcare accessibility in European countries.
- Articles published in peer-reviewed journals between 2020 and 2024.
- Research articles, systematic reviews, and scoping reviews written in English.

Exclusion Criteria:

- Letters, editorials, and opinion pieces.
- Conference abstracts and proceedings.
- Grey literature (e.g., government reports, unpublished theses).
- Studies not explicitly addressing healthcare accessibility.
- Studies not conducted in European countries.
- Studies for which full-text access was unavailable.

The review was limited to studies published between 2020 and 2024 in order to capture the most recent developments in GIS applications for healthcare accessibility, particularly those influenced by the COVID-19 pandemic and subsequent innovations in methodological approaches.

### 2.5. Study Selection

**Figure 1:**
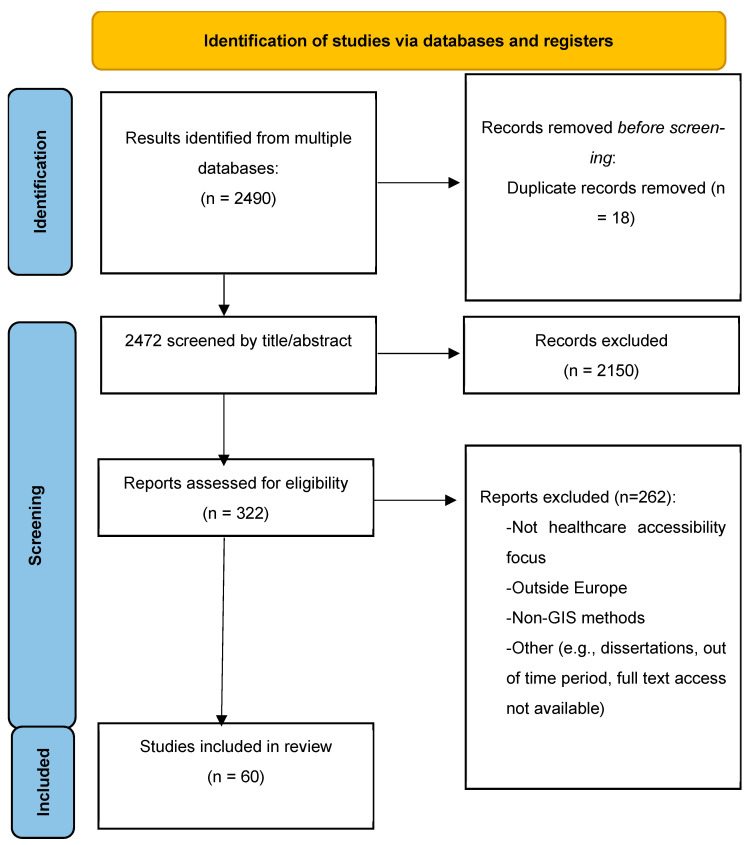
Search and study selection.

The database search identified 2490 records. After removing 18 duplicates in Rayyan.ai (https://www.rayyan.ai/), 2472 records were screened by title and abstract. Of these, 2150 were excluded. The full text of 322 articles was then assessed for eligibility, leading to the exclusion of 262 articles for reasons such as lack of focus on healthcare accessibility, being conducted outside Europe, absence of GIS-based methods, or other methodological limitations. Ultimately, 60 studies met all inclusion criteria and were included in the final synthesis (Figure 1). Any discrepancies between reviewers during screening were resolved through discussion until consensus was reached.

### 2.6. Data Charting

To facilitate the summary and analysis of the included studies, an Excel table was created by one author (T.A.), which contained key information for each study, including purpose, study area, population served, use of GIS, type of GIS tool, methods, results and limitations. After the initial extraction of data, a second author (S.N.) cross-checked the information to ensure accuracy and consistency in reporting. The charting framework was developed in advance based on the PCC structure and piloted on a small subset of studies before full extraction.

### 2.7. Summary and Reporting of Results

Two reviewers analysed the data from the selected studies. Initially, the reviewers familiarised themselves with the content of the articles to ensure a comprehensive understanding of each study’s approach and findings. Then, the key findings reported in the articles were extracted and systematically charted in a data table [11,12,13,15,19,22,23,24,25,26,27,28,29,30,31,32,33,34,35,36,37,38,39,40,41,42,43,44,45,46,47,48,49,50,51,52,53,54,55,56,57,58,59,60,61,62,63,64,65,66,67,68,69,70,71] (Table S1). The studies highlighted key factors influencing healthcare access, including proximity to healthcare facilities, transportation networks, and geographic disparities. Detailed information on these findings was organised in a data table for clarity and ease of comparison (see Table S1). The analysis was conducted through manual thematic synthesis of the charted data, without software assistance, to identify recurring themes and patterns across studies.

## 3. Results

### 3.1. Study Characteristics

**Figure 2:**
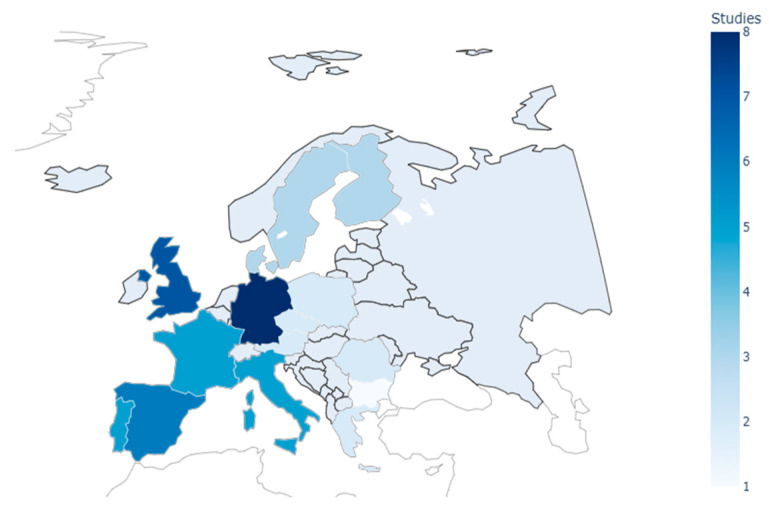
Distribution of reviewed studies by European country.

Sixty studies conducted across 20 European countries were included in this review, with the majority originating from Western and Southern Europe (Table S1). In contrast, research from Eastern European countries was comparatively scarce (Figure 2). Twenty-eight studies targeted the general population, while 32 examined specific demographic groups, including older adults [13,43,44,45,46,47,72,73], patients with chronic conditions [48,49,50,51,52,53,54], individuals requiring rehabilitation [55,56,57,58], rural and remote communities [59,60,61], mobility-limited patients [62,63], cross-border users [64,65], and populations experiencing structural barriers [66,67,68,69,70]. All studies employed GIS-based methods, ranging from descriptive spatial mapping to advanced modelling approaches. The predominance of research from Western European contexts reflects a geographical imbalance in the evidence base and highlights the limited coverage of Eastern regions, where health system challenges remain comparatively underexplored.

### 3.2. Methodological Applications

The included studies demonstrated a broad spectrum of GIS-based approaches to evaluating healthcare accessibility, which clustered into four main categories: (1) floating catchment area (FCA) methods, (2) transport-modality approaches, (3) advanced spatial analytics, and (4) hybrid decision-support frameworks. Each of these methodological families was applied in distinct ways across European contexts, reflecting both regional priorities and variations in data availability.

Floating catchment area (FCA) methods were the most frequently employed. The two-step FCA (2SFCA) and its enhanced variants were commonly used to calculate provider–population ratios and travel times. Bauer et al. [23], for instance, assessed intensive care units across 14 European countries and reported mean car travel times of 13.1 min, with values ranging from 9.1 min in Luxembourg and 9.3 min in Germany to 25.3 min in Croatia. Other refinements extended FCA applications to specific contexts: the modified Huff three-step FCA (MH3SFCA) revealed rural–urban differences and seasonal demand shifts in Germany’s tourist-intensive regions [40]. In Poland, 2SFCA methods identified primary-care “deserts” in border areas through road-network analysis [12], while studies in Germany, Austria, and the Czech Republic applied FCA approaches to secondary and outpatient services [15,38,74].

Transport-modality approaches accounted for nearly one-third of the reviewed studies and reflected strong regional tailoring [11,13,33,39,73]. In Finland, kotavaara et al. [11] combined road networks with General Transit Feed Specification (GTFS) data at a 250 m grid resolution to demonstrate disparities in healthcare access between car-owning and carless households. Mediterranean studies often prioritised pedestrian accessibility: Carpentieri et al. [13] modelled primary-care access in Naples using reduced walking speeds for adults aged 75 years and older (3.5 km/h), while Portuguese researchers integrated disability-adjusted walking times to capture accessibility barriers among mobility-impaired patients [73]. In Scandinavia, emergency service models incorporated helicopter thresholds, such as the 29 min response standard for Danish island populations without road connections [39]. Similar multimodal analyses have been used in Sweden and Portugal to assess hospital and emergency care coverage [27,33].

Advanced spatial analytics provided critical evidence of structural inequities and their health implications [25,43,50,66]. In Spain, spatial autocorrelation (Moran’s I) revealed significant clusters of asthma-related admissions, with smoothed relative risk values exceeding 150 across northern municipalities compared to values of ≤65 in southern areas [50]. In France, geographically weighted regression showed that each additional general practitioner per km^2^ was associated with a 1.2-day reduction in average hospital stay [43]. Kernel density estimation identified underserved “cold spots” for spa-based rehabilitation in rural France [25]. Other applications, such as cluster analyses in Central and Eastern Europe [66], further demonstrated how geostatistical techniques could capture inequalities not readily visible through traditional accessibility measures.

**Figure 3:**
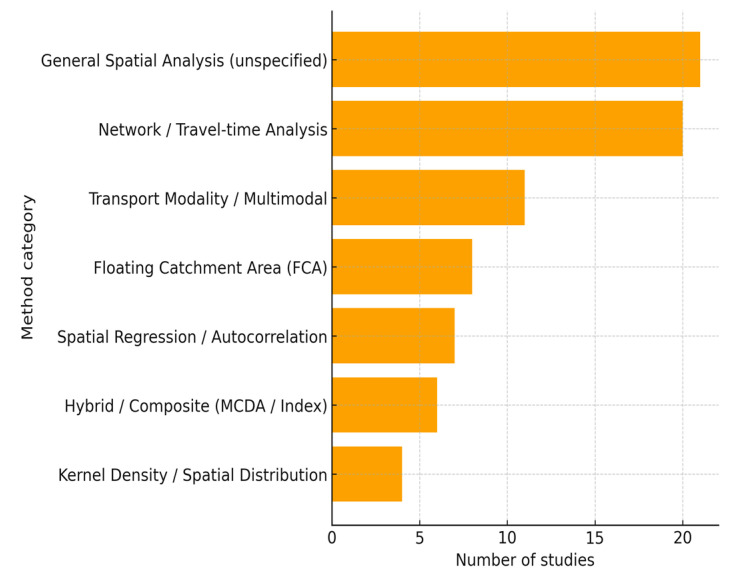
GIS tools and techniques used in the reviewed studies.

Hybrid decision-support frameworks marked the most innovative strand of methodological development. In Portugal, Lopes et al. [37] integrated multi-criteria decision analysis (UTASTAR) with GIS to evaluate hospital networks across seven weighted dimensions. In France, the SCALE index merged four spatiotemporal parameters to create a composite measure of national primary-care accessibility [36]. In Germany, temporal modelling of day- and night-time populations showed that stroke-care coverage was 18.1% lower at night compared to daytime [56], highlighting the added value of incorporating dynamic population shifts into accessibility analyses (Figure 3).

Despite their increasing sophistication, these methodological applications exhibited several recurring limitations. While some studies validated their models against healthcare utilisation data [43,48,50], only a small number accounted for temporal variations in population distribution [56,60] or incorporated socioeconomic determinants of access [29,30]. The absence of standardised methods across countries further restricted comparability and hindered the development of a coherent evidence base.

### 3.3. Regional Disparities

**Figure 4:**
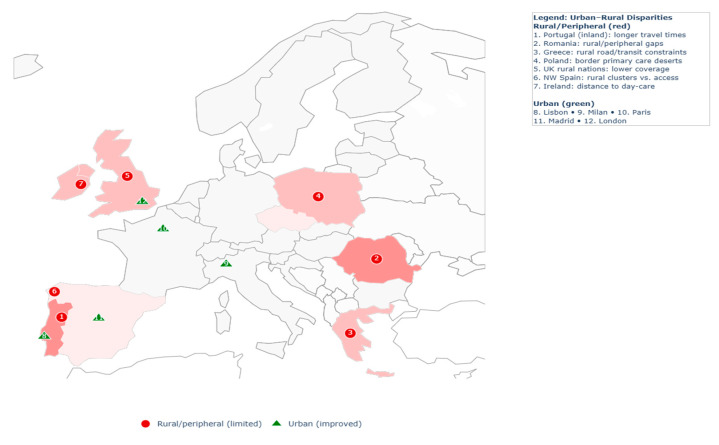
Urban–rural disparities in healthcare accessibility.

The reviewed studies identified spatial disparities in healthcare accessibility across European contexts, most notably between urban and rural areas, but also within metropolitan regions and across national borders (Figure 4). These disparities revealed persistent inequalities in service provision despite overall improvements in healthcare infrastructure.

Urban–rural divides were the most frequently reported. In Portugal, national hospital accessibility improved by approximately 10% over two decades; however, inland municipalities and border regions continued to experience significantly longer travel times than coastal areas [27]. In Ireland, almost one-fifth of people with dementia lived more than 15 km from the nearest day-care centre [48]. Similar patterns were observed in the United Kingdom, where only 18–40% of residents in rural Wales, Scotland, and Northern Ireland had access to a dental practice within 2.5 km, compared to near-universal coverage in urban settings [75]. In Poland, 2SFCA analysis revealed that high population density did not necessarily translate into better accessibility, as primary care provision remained constrained in border regions [12]. Comparable inequalities were documented in Romania and Greece, where shortages of physicians and uneven facility distribution disproportionately affected rural communities [29,32].

Disparities were also observed within cities. In Milan, older adults living in peripheral neighbourhoods had markedly poorer access to primary healthcare compared to those in central areas [44]. In France, municipalities characterised by higher socioeconomic deprivation clustered spatially with increased mortality rates, suggesting that access barriers may exacerbate broader health inequities [30].

Peripheral and cross-border regions exhibited additional disadvantages. Studies from Central and Eastern Europe reported reduced access to specialised services such as intensive care and emergency provision in border areas [38,64]. These findings indicated that structural disadvantages persisted even where national averages suggested adequate coverage.

Regional disparities in healthcare accessibility thus remained a defining feature across European contexts. While urban centres benefitted from dense service networks, rural, peripheral, and socioeconomically disadvantaged areas were consistently identified as underserved, reflecting enduring structural patterns insufficiently addressed by existing healthcare provision.

### 3.4. Transportation as a Determinant of Accessibility

Transportation systems exerted a critical influence on healthcare accessibility, functioning as both an enabling and constraining factor across European contexts. Rural and peripheral regions were disproportionately affected by weak infrastructure, with studies from Greece and Romania showing that poor road conditions and limited public transit substantially prolonged access times despite nominal proximity to facilities [29,32]. Analyses in Portugal further demonstrated that households without private vehicles faced markedly higher travel burdens, underlining the structural dependence of service access on car ownership [27].

In metropolitan areas, the interaction of transport conditions with demographic vulnerabilities intensified inequities. In Paris, traffic congestion extended emergency travel times in socioeconomically disadvantaged districts [30], while in Milan, reduced pedestrian mobility among older adults accentuated accessibility gaps between central and peripheral neighbourhoods [44]. These findings illustrate how transport-related barriers compound existing social and spatial inequalities within urban systems.

**Figure 5:**
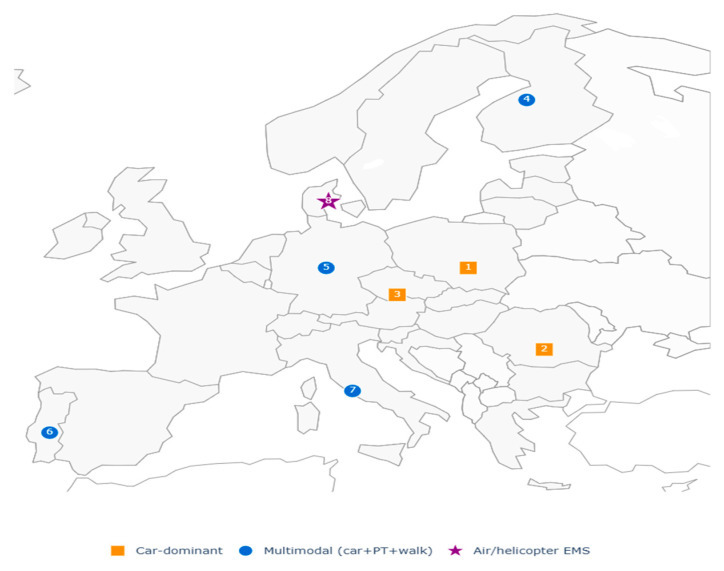
Transportation-related accessibility approaches in reviewed studies. Colors indicate the type of transport-related GIS method, whereas numbers (1–8) represent the number of studies in the corresponding geographic areas that applied each specific approach.

Transport challenges were particularly evident in emergency and cross-border contexts. In Denmark’s island regions, reliance on helicopter medical services was indispensable to achieving national response standards, with median travel times approaching 29 min in areas without road connections [39]. Populations in Central Europe similarly encountered extended delays where cross-border service integration was limited, reflecting the combined impact of physical and administrative mobility constraints [64] (Figure 5).

Reviewed studies demonstrated that mobility barriers were a consistent contributor to geographic and social inequities. However, few investigations explicitly compared transport systems across countries or examined how long-term infrastructure investments altered access patterns over time. These gaps indicate that transportation remains an underexplored yet decisive dimension of healthcare accessibility.

### 3.5. Accessibility and Healthcare Utilisation

Only a small number of studies examined links between accessibility and healthcare utilisation, most relying on indirect comparisons of modelled access indicators with service demand proxies. In France, higher densities of general practitioners were linked to shorter average hospital stays, suggesting that primary care availability can reduce reliance on inpatient services [43]. In Spain, clusters of asthma-related admissions overlapped with municipalities reporting lower accessibility scores, reflecting a misalignment between healthcare needs and provision [50]. Comparable results were documented in Poland and Portugal, where underserved populations depended more on emergency services and experienced delays in routine care [12,27]. A smaller group of studies validated accessibility measures against utilisation data, showing that longer travel times were associated with fewer outpatient visits and lower rehabilitation uptake [48,55]. Such validations, however, were rare and employed heterogeneous approaches, limiting comparability across contexts.

The reviewed studies illustrate that the use of GIS-based assessments to approximate utilisation patterns remains limited, characterised by few validation efforts, methodological heterogeneity, and restricted cross-national comparability, and therefore lacks the consistency required to support broader generalisations.

### 3.6. Policy and Planning Applications

A subset of studies explicitly framed GIS analyses as instruments for healthcare planning. In Portugal, the integration of multi-criteria decision analysis with GIS enabled the evaluation of hospital networks across seven weighted dimensions, offering a structured basis for policy prioritisation [37]. In France, the SCALE index combined spatiotemporal parameters into a composite primary care accessibility score designed for national monitoring and planning [36]. Temporal population modelling in Germany revealed an 18.1% reduction in night-time stroke-care coverage compared with daytime, emphasising the importance of dynamic population data in emergency planning [56]. In Denmark, the incorporation of helicopter thresholds into accessibility assessments ensured compliance with national response standards for island populations [39]. Other applications addressed sector-specific needs. Kernel density mapping in rural France identified rehabilitation “cold spots,” providing an evidence base for regional service expansion [25]. Cross-border analyses in Central Europe demonstrated how administrative and infrastructural barriers restricted service access, with direct implications for EU-level coordination of specialised care [64].

The studies that addressed planning applications demonstrated varied uses of GIS, ranging from network evaluation and national monitoring to emergency preparedness and cross-border coordination. However, such applications were relatively few, relied on heterogeneous approaches, and offered only partial evidence of how GIS has been systematically embedded in healthcare planning across Europe.

## 4. Discussion

This scoping review examined the application of Geographic Information Systems in healthcare accessibility research across European countries between 2020 and 2024. The findings demonstrate that GIS has become a versatile instrument for measuring spatial inequities, identifying underserved populations, and informing healthcare planning. Importantly, the studies reviewed illustrate how GIS enables the quantification of disparities that may otherwise remain obscured within aggregate health statistics, thereby contributing a distinctive spatial perspective to the evidence base on healthcare inequalities. At the same time, considerable variation in methodological approaches, regional coverage, and thematic priorities continues to limit the comparability of findings and constrain their systematic translation into policy and practice.

A prominent theme emerging from the literature is the persistence of geographical differences in healthcare accessibility. Rural and peripheral regions were repeatedly characterised by longer travel times, weaker transport infrastructure, and reduced availability of services, reflecting enduring barriers even where national healthcare systems had undergone broader improvements [76,77]. In urban contexts, socioeconomic deprivation compounded spatial disadvantage, as lower-income neighbourhoods often faced constrained access to facilities located in close proximity [78]. These patterns emphasise the role of accessibility as both a spatial and structural determinant of health and highlight the need for equity-oriented planning strategies that address imbalances in service provision [71,79].

The adoption of GIS has been most advanced in Western Europe, particularly in the United Kingdom, Germany, Spain, and the Netherlands, where strong health information infrastructures, substantial investment in spatial technologies, and established expertise have facilitated sophisticated analyses of accessibility [80,81]. By contrast, Eastern European countries continue to face challenges related to fragmented health information systems, limited availability of spatial data, and a shortage of trained specialists [82]. This regional imbalance points to an uneven capacity for incorporating GIS into healthcare planning, with implications for the comparability of evidence across contexts and the ability of less-resourced health systems to systematically address accessibility inequities.

Methodologically, the reviewed studies revealed both innovation and fragmentation. Floating catchment area models, particularly two-step and enhanced variants, were most frequently applied to estimate provider-to-population ratios and travel times. These approaches produced detailed insights into spatial distribution but were also sensitive to data quality and local system configurations, which complicates comparative analysis across different settings [81,83]. Recent scholarship has suggested that methodological advancement will require integration of emerging techniques such as machine learning, real-time mobility data, and longitudinal monitoring, which could enhance both the precision and policy relevance of GIS analyses [84].

Transportation was consistently identified as a decisive factor shaping healthcare accessibility. Populations without private vehicles, older adults, and individuals with mobility limitations were disproportionately affected in contexts where public transport networks were weak or fragmented. Studies employing multimodal GIS approaches—which integrated car use, public transport, and pedestrian mobility—demonstrated the value of incorporating transport heterogeneity into accessibility assessments, thereby generating more comprehensive accounts of the barriers faced by vulnerable groups [85,86,87,88,89,90,91]. Such findings highlight the importance of embedding transport considerations more systematically into healthcare planning frameworks, particularly in regions where reliance on single-mode analysis risks masking underlying inequities.

The relationship between spatial accessibility and healthcare utilisation remains comparatively underdeveloped within the evidence base. A limited number of studies suggested that proximity to providers and higher provider density were associated with increased uptake of preventive and primary care, while underserved areas reported delays in treatment initiation or greater reliance on emergency services. Yet few attempts were made to validate accessibility models against observed utilisation data, and where such efforts occurred, they employed heterogeneous measures and analytic techniques, limiting comparability and the potential for generalisation [22,92,93,94,95]. Strengthening this line of inquiry will depend on the systematic integration of utilisation outcomes into spatial analyses, alongside the development of standardised indicators capable of capturing both temporal and equity-sensitive dimensions of service use.

The uneven adoption of GIS across Europe reflects barriers that are institutional and structural rather than technological. Frequently cited obstacles included restricted access to reliable health and spatial data, absence of methodological standardisation, resource constraints, and insufficient institutional or political support [96,97]. Addressing these barriers will require sustained collaboration among policymakers, healthcare organisations, and the research community to establish common protocols for GIS-based accessibility assessments and to expand capacity through training, infrastructure investment, and integration of health and spatial data systems [98,99]. By building stronger foundations for GIS application, European health systems will be better positioned to translate spatial analyses into evidence-informed strategies that reduce disparities and support more equitable service provision.

Finally, several limitations of this review should be noted. The exclusion of non-English publications may have led to the omission of studies from regions where English is not the primary academic language. The focus on peer-reviewed work published between 2020 and 2024 may also have introduced publication bias by excluding earlier or grey literature. Moreover, the heterogeneity of GIS methodologies complicates synthesis and constrains cross-country comparability. The relative underrepresentation of Eastern European contexts further reduces the extent to which findings can be extrapolated across the continent, underscoring the importance of expanding the geographical scope of future research.

## 5. Conclusions

This review positions Geographic Information Systems as an indispensable analytical framework for investigating healthcare accessibility in Europe, offering spatial perspectives that uncover inequities frequently obscured within conventional health statistics. The synthesis demonstrates that although floating catchment area models and network analyses have become prevalent methodological approaches, their applications consistently reveal persistent urban–rural divides, transportation barriers, and pronounced contrasts between Western and Eastern Europe. These disparities are not merely geographical but deeply structural, rooted in unequal infrastructure development, fragmented health information systems, and disparities in institutional capacity and investment in geospatial technologies across the continent.

Despite the demonstrated promise of GIS for advancing equity in healthcare planning, several substantive limitations remain. The limited validation of accessibility models against empirical utilisation data, the considerable methodological heterogeneity across studies, and the underrepresentation of Eastern and Southeastern Europe collectively constrain cross-national comparability and diminish policy relevance. These gaps highlight the urgent need for harmonised methodological frameworks that integrate spatial, demographic, and socioeconomic indicators, enabling a more nuanced and comprehensive understanding of healthcare accessibility in diverse contexts.

Future research should progress beyond descriptive mapping toward the systematic integration of longitudinal geospatial data, transport and equity indicators, and advanced analytic techniques such as artificial intelligence, real-time mobility data, and machine-learning-based spatial modelling. Advancing along these lines will enhance methodological robustness, elucidate the structural mechanisms underlying healthcare inequities, and strengthen the capacity of GIS to inform evidence-based, equity-oriented policy and planning across Europe’s heterogeneous healthcare systems.

## Figures and Tables

**Table 1:** Population, concept and context of the study.

Population	Human participants in European countries; studies conducted on accessibility to healthcare and health services.
Concept	Use of Geographic Information System (GIS) analysis for healthcare access studies conducted from 2020 to 2024.
Context	Access to healthcare services, including healthcare facilities and health services in European countries.

## Data Availability

No new data were created or analysed in this study. Data sharing is not applicable to this article as it is based on a review of existing literature.

## Supplementary Materials

The following supporting information can be downloaded at https://www.mdpi.com/article/10.3390/healthcare13222865/s1: Table S1. Results of the scoping review.

## Abbreviations

The following abbreviations are used in this manuscript:

GIS	Geographic Information Systems
PCC	Population, Concept, and Context
FCA	Floating catchment area
2SFCA	Two-step floating catchment area
ICU	Intensive care unit
MH3SFCA	Modified huff three-step floating catchment area
E2SFCA	Enhanced wo-step floating catchment area
GTFS	General Transit Feed Specification
EMS	Emergency medical services
GWR	Geographically weighted regression
MCDA	Multiple Criteria Decision Analysis
EU	Europe/European
UK	United Kingdom
